# Supplementary material for: A Critical View of the Application of the APEX Software (Aqueous Photochemistry of Environmentally-Occurring Xenobiotics) to Predict Photoreaction Kinetics in Surface Freshwaters
Source: Molecules. 2019 Dec 18;25(1):9. doi: 10.3390/molecules25010009 (PMC7017383; doi:10.3390/molecules25010009)
Supplement: Supplementary file 1 [file molecules-25-00009-s001.zip › 3.2.4_gcc-4.4.0.zip!/3.2.4_gcc-4.4.0/3.2.4_gcc-4.4.0/doc/octave/html/interpreter/Diary-and-Echo-Commands.html]

Diary and Echo Commands - Untitled


Previous: Customizing the Prompt,
Up: Command Line Editing


---

#### 2.4.8 Diary and Echo Commands

Octave's diary feature allows you to keep a log of all or part of an
interactive session by recording the input you type and the output that
Octave produces in a separate file.

— Command: **diary** options  
> Record a list of all commands *and* the output they produce, mixed
> together just as you see them on your terminal. Valid options are:
>
> `on`: Start recording your session in a file called diary in your current working directory. `off`: Stop recording your session in the diary file. file: Record your session in the file named file.
>
> With no arguments, `diary` toggles the current diary state.

Sometimes it is useful to see the commands in a function or script as
they are being evaluated. This can be especially helpful for debugging
some kinds of problems.

— Command: **echo** options  
> Control whether commands are displayed as they are executed. Valid
> options are:
>
> `on`: Enable echoing of commands as they are executed in script files. `off`: Disable echoing of commands as they are executed in script files. `on all`: Enable echoing of commands as they are executed in script files and functions. `off all`: Disable echoing of commands as they are executed in script files and functions.
>
> With no arguments, `echo` toggles the current echo state.

— Built-in Function: val = **echo\_executing\_commands** ()  
— Built-in Function: old\_val = **echo\_executing\_commands** (new\_val)  
> Query or set the internal variable that controls the echo state.
> It may be the sum of the following values:
>
> 1: Echo commands read from script files. 2: Echo commands from functions. 4: Echo commands read from command line.
>
> More than one state can be active at once. For example, a value of 3 is
> equivalent to the command `echo on all`.
>
> The value of `echo_executing_commands` may be set by the `echo`
> command or the command line option `--echo-commands`.
